# METTL3/IGF2BP2 Promotes the Malignant Progression of Esophageal Cancer by Activating the PIK3CA/AKT Pathway

**DOI:** 10.1111/1759-7714.70022

**Published:** 2025-02-20

**Authors:** Xinmeng Guo, Anqi Huang, Ya'nan Qi, Jiaqi Chen, Meng Yang, Mulan Jin

**Affiliations:** ^1^ Department of Pathology Beijing Chao‐Yang Hospital, Capital Medical University Beijing China

**Keywords:** AKT, esophageal cancer, IGF2BP2, METTL3, PIK3CA

## Abstract

Esophageal cancer (EC) is a leading cause of cancer‐related mortality worldwide. Methyltransferase‐like 3 (METTL3), a key enzyme involved in m6A methylation, has been implicated in the development and progression of various cancers, including EC. However, its potential mechanism of action in EC progression remains unclear. METTL3 expression was found to be upregulated in EC tissues and cells. Knockdown of METTL3 suppressed EC cell proliferation, invasion, migration, and angiogenesis, while promoting apoptosis. Mechanistically, METTL3 maintained PIK3CA mRNA expression and stability in an m6A‐dependent and IGF2BP2‐dependent manner, respectively. METTL3 silencing inactivated the AKT pathway by regulating PIK3CA expression. Furthermore, overexpression of PIK3CA mitigated the effects of METTL3 silencing on the malignant growth of KYSE180 and TE1 cells in vivo and in vitro. METTL3/IGF2BP2 promoted the malignant progression of EC by activating the PIK3CA/AKT pathway. Targeting the METTL3‐PIK3CA axis may offer a novel therapeutic approach for EC treatment.

## Graphical abstract

METTL3 induced PIK3CA expression by an m6A‐IGF2BP2‐dependent mechanism, and the elevation in PIK3CA expression activated the AKT pathway, thus promoting EC cell proliferation, migration, invasion, and angiogenesis and inhibiting cell apoptosis.

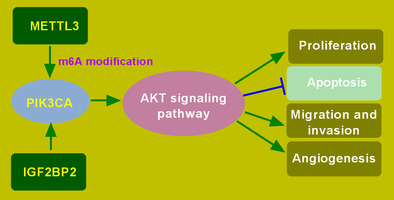

## 1 Introduction

Esophageal cancer (EC) is a prevalent and aggressive type of digestive system malignancy and is primarily categorized into two pathological subtypes [1]. The pathogenesis of EC is complex and closely associated with genetic factors, environmental influences, and lifestyle habits. With advancements in screening techniques, early‐stage EC can be effectively treated with endoscopic procedures based on the depth of invasion, improving prognosis and reducing recurrence rates [2]. For locally advanced, unresectable cases with lymph node metastasis, a combination of surgery, radiotherapy, chemotherapy, and targeted immunotherapy has become the standard treatment for locally advanced ESCC [3]. Despite progress in diagnostic techniques and treatment methods for ESCC, the overall prognosis for EC remains poor, with a current 5‐year survival rate of less than 30% [4]. Therefore, further investigation into the mechanisms underlying EC is crucial for identifying potential therapeutic targets for intervention.

In recent years, N6‐methyladenosine (m6A) RNA modification has been pushed to the forefront of bioscience, with its dynamic and reversible process being capable of regulating different gene expressions and biological functions. The m6A modification is installed by “writers” and removed by “erasers” in eukaryotic cells, and its presence is detected by “readers,” which then initiate downstream signaling pathways [5]. Recent studies have shown that m6A modification has become a new layer of regulatory mechanism for disease pathogenesis and treatment response [6, 7]. METTL3 is a methyltransferase with a full length of 580 amino acids, consisting of a zinc finger domain (ZFD) and a methyltransferase domain, both of which are essential for enzyme activity [8, 9]. Increasing evidence in recent years has shown that METTL3 participates in tumor cell proliferation, metastasis, and tumor microenvironment [10]. In particular, METTL3 plays a cancer‐promoting role in EC progression [11]; however, the specific mechanism remains unclear.

The enzyme phosphoinositide 3‐kinase (PI3K) encodes the p110α catalytic subunit, a key component of the PI3K complex [12]. The p110α catalytic subunit of Class I PI3K is encoded by the PIK3CA gene, which spans 86 190 base pairs across 21 exons and results in a transcript of 3207 base pairs, coding for a protein of 1068 amino acids [13]. Somatic mutations in the PIK3CA gene are commonly described in human cancers, such as colorectal cancer [14] and lung cancer [15]. In primary breast cancer, the prevalence of PIK3CA mutations can reach as high as 40% [16]. The most frequent mutations in the PIK3CA gene occur in coding sequences that lead to a gain‐of‐function in PI3K activity. Previous evidence has indicated that METTL3 downregulates miR‐320a expression to promote PIK3CA expression in osteomyelitis progression [17]. In particular, PIK3CA overexpression was involved in the lymph node metastasis of EC patients [18]; however, whether METTL3 promotes EC progression by stabilizing PIK3CA remains known.

Based on the above evidence, the study proposed that METTL3 induced the m6A methylation of PIK3CA and thus stabilized PIK3CA expression, further promoting the malignant progression of EC cells. Through a series of experiments, the study validated this hypothesis, with the ultimate goal of identifying a novel therapeutic target for the treatment of EC.

## 2 Materials and Methods

### 2.1 Cell Culture

EC cell lines (including KYSE30, TE‐1, and KYSE150) and human esophageal epithelial cells (HEECs) were provided by Procell (Wuhan, China). EC cells (KYSE‐180) and human umbilical vein endothelial cells (HUVECs) were provided by EK‐Bioscience (Shanghai, China). HUVECs were cultured in DMEM (Procell). KYSE30 cells were cultured in RPMI‐1640/Ham's F‐12 medium (Procell) containing 2 mM l‐Glutamine (Sigma, St. Louis, MO, USA), KYSE150 cells were maintained in RPMI‐1640/Ham's F‐12 medium, and other cells were cultured in RPMI‐1640 medium (Procell). In addition, cell culture was performed in these media added with 10% fetal bovine serum (Procell) and 1% penicillin/streptomycin (Procell) at 37°C with 5% CO_2_.

### 2.2 Cell Transfection

Small interfering RNAs specific to METTL3 (si‐METTL3#1 and si‐METTL3#2) and IGF2BP2 (si‐IGF2BP2), PIK3CA overexpression plasmid (OE‐PIK3CA), and the matched controls (si‐NC, OE‐NC) were provided by Ribobio Co. Ltd. (Guangzhou, China). The mixtures of Opti‐MEM (Worbisen Technology, Beijing, China) and FuGENE6 (Roche, Basel, Switzerland) or siRNAs/plasmids were prepared and incubated for 20 min. The mixtures were added to culture cells, and the cells were cultured for the following assays.

### 2.3 Quantitative Real‐Time Polymerase Chain Reaction (qRT‐PCR)

The tissues treated with liquid nitrogen or cells collected by centrifugation were treated with TRIzol reagent (TaKaRa, Dalian, China) for lysis, followed by centrifugation to extract RNA. The extracted RNA, prime Script cDNA synthesis reagents (TaKaRa), and RNase‐free water were mixed according to the instructions to prepare the reverse transcription reaction mixtures. The mixtures were then subjected to reverse transcription in a PCR machine. The cDNA, target gene primers, SYBR Premix (TaKaRa), and RNase‐free water were mixed according to the instructions to prepare the quantification reaction mixture. The amplification was carried out using a two‐step method. GAPDH and U6 were used as internal controls, and target genes were quantitatively calculated using the 2^−ΔΔCT^ method. METTL3 5′‐CAGAGGCAGCATTGTCTCCA‐3′ and 5′‐ATGGACACAGCATCAGTGGG‐3′, PIK3CA 5′‐GGACCCGATGCGGTTAGAG‐3′ and 5′‐ATCAAGTGGATGCCCCACAG‐3′, 5′‐AGAAGGCTGGGGCTCATTTG‐3′, and 5′‐AGGGGCCATCCACAGTCTTC‐3′.

### 2.4 Western Blotting Assay

Protein samples were prepared using RIPA buffer (Beyotime, Shanghai, China) and mixed with loading buffer (Beyotime). Electrophoresis was performed, and membranes were incubated with primary antibodies specific to METTL3 (1:1000, Affinity, Nanjing, China), PIK3CA (1:1000, Affinity), IGF2BP2 (1:1000, Affinity), p‐AKT (1:1000, Quayad, Beijing, China), AKT (1:1000, Quayad), and GAPDH (1:8000, Quayad). The protein membrane was placed in the pre‐prepared secondary antibody solution (1:5000, Affinity) and then incubated on a shaker. Finally, the hypersensitive ECL luminescent solution (Beyotime) was used to detect the protein bands.

### 2.5 Bioinformatics Analysis

The GEO database GSE254232 (https://www.ncbi.nlm.nih.gov/geo/query/acc.cgi?acc=GSE254232) was used to analyze the differently expressed genes in EC cells (EC109), with a *P* value < 0.00001 and an absolute log_2_FC > 2. Through the TCGA database (https://ualcan.path.uab.edu/analysis.html) and the TNMplot database (https://tnmplot.com/analysis/), METTL3 and PIK3CA expression were analyzed in EC and normal esophageal tissues. By using the RMbase database (https://rna.sysu.edu.cn/rmbase3/modgene.php), the target gene PIK3CA was input to detect whether the gene has m6A modification. The mRNA sequence of PIK3CA was retrieved through NCBI (https://www.ncbi.nlm.nih.gov/nuccore/NM_006218.4), and the SRAMP website (http://www.cuilab.cn/sramp/) was used to input the mRNA sequence of PIK3CA to analyze m6A modification sites on its mRNA.

### 2.6 Cell Viability Analysis

Dilution of the cell suspension based on the seeding concentration was carried out, followed by placement of the cells in a 37°C environment for cultivation for 48 h. Cell counting kit‐8 (CCK‐8) solution (Beyotime) was directly added to each well, and the culture plates were placed for 2–4 h. The OD values were measured in each well using a microplate reader.

### 2.7 Cell Proliferation Analysis

Cells were digested and seeded into 96‐well plates and incubated overnight. 5‐Ethynyl‐2′‐deoxyuridine (EdU, 10 mM, Ribobio) diluted with complete medium was added to each well. The cells were further incubated for an appropriate amount of time (2–4 h). 4% paraformaldehyde was added to fix the cells. Click reaction mixture (Ribobio) and DAPI solution (Ybiotech, Shanghai, China) were added to each well. After incubation in the dark, the cells were observed under a fluorescence microscope. The cell nuclei appeared as blue fluorescence, while proliferating cells displayed bright green fluorescence.

### 2.8 Cell Apoptosis Analysis

Cells were digested using trypsin (Khayal Biotechnology, Wuhan, China) without EDTA and transferred to centrifuge tubes, where they were centrifuged to collect the cell pellets. The cell pellets were resuspended in 1 × binding buffer (Solarbio). A total of 1 × 10^5^ cells were drawn into centrifuge tubes, to which 5 μL of Annexin V‐FITC (Solarbio) and 10 μL of propidium iodide (Solarbio) were added. After gentle mixing, the cells were incubated in the dark for 15 min. Following staining and incubation, 1 × binding buffer working solution was added, and the mixture was thoroughly mixed. Flow cytometry analysis was performed within 1 h.

### 2.9 Transwell Invasion Assay

Cells were resuspended in a serum‐free medium to create a cell suspension. The lower chambers of the transwell inserts were filled with a complete medium containing 20% FBS, and 3 × 10^4^ cells were seeded into the upper chambers coated with Matrigel (Abwbio, Shanghai, China). The inserts were then placed in a cell culture incubator and incubated for 24 h. After removal from the incubator, the liquid in the upper chamber was wicked away with a cotton swab, and the inserts were placed into new 24‐well plates. To fix the cells, 700 μL of 4% paraformaldehyde (Sangon Biotech, Shanghai, China) was added to the lower chamber. Subsequently, 700 μL of 0.1% crystal violet (Sigma) was added to the lower chambers for 20 min. The invasion of cells was observed under a microscope, and photographs of five random fields of cells were taken.

### 2.10 Wound‐Healing Assay

Three parallel black lines were drawn on the back of each well in six‐well plates in advance using a marker pen. EC cells were seeded into each well and incubated in a cell culture incubator overnight. After the cells had completely covered the bottom of the wells, a 10‐μL pipette tip was used to make parallel scratches across the cell monolayer, aligning with the black lines at the bottom of the wells. The culture medium was removed, and serum‐free medium was added to each well. Photographs were taken under a microscope to document the scratches at 0 h. After 24 h of further incubation, the changes in the scratch area were observed, and photographs were taken from the same position to compare the changes in the area of the scratched region.

### 2.11 Tube Formation Assay

About 200 μL of growth factor‐depleted Matrigel (Abwbio) was added to each well of 24‐well plates and placed in a 37°C incubator to settle for 30 min. After the gel had solidified, HUVEC cells were added to the wells at a concentration of 1 × 10^4^ cells per well. EC cell‐conditioned medium was then added to the culture plates, and the plates were gently swirled before being incubated for 4–6 h. Subsequently, the tubular structures were observed and photographed under a microscope, and their lengths and numbers were recorded to analyze the angiogenic capacity of HUVECs.

### 2.12 m6A RNA Immunoprecipitation (MeRIP) Assay

m6A MeRIP Kit (BersinBio, Guangzhou, China) was used for the assay. In brief, EC cells were added to premixed lysis buffer, which was then placed on ice for 10 min. The mixture was centrifuged, and the cell lysate samples were divided into two groups: the IgG antibody group and the m6A antibody group. Experimental antibodies were added, and the samples were mixed vertically and incubated overnight at 4°C. The magnetic beads were collected using a magnetic rack. The two groups of samples were then incubated at 55°C for 1 h to elute RNA. The magnetic beads were collected again using a magnetic rack, and the supernatant was transferred to new enzyme‐free centrifuge tubes. RNA was extracted, and qRT‐PCR was performed to validate the binding efficiency of METTL3 for PIK3CA.

### 2.13 RNA Immunoprecipitation (RIP) Assay

Cells at approximately 90% confluence were collected and resuspended in RIPA lysis buffer (Beyotime). Protein A/G agarose beads (Millipore) were incubated with the antibody specific to METTL3 (1:100, Thermo Fisher), IGF2BP2 (1:800, Thermo Fisher) or IgG (1:100, Abcam). The complexes were washed, and then the Protein A/G complexes were resuspended in Proteinase K buffer. RNA was extracted and subjected to reverse transcription and qRT‐PCR analysis for PIK3CA expression detection.

### 2.14 Actinomycin D Assay

The cells from the experimental and control groups were seeded in appropriate amounts into 12‐well plates and labeled as 0, 3, and 6 h, respectively. The cells were incubated in a cell culture incubator for 4–6 h to allow them to adhere to the plates. After 6 h, 1 μL of actinomycin D solution (Abcam) was added to the 6 h group to achieve a final concentration of 10 μM in the culture medium, and the incubation was continued for an additional 3 h. Then, 1 μL of actinomycin D solution was added to the 3 h group, and the incubation was continued for another 3 h. RNA was extracted from each well, and qRT‐PCR was performed to validate the inhibition efficiency of IGF2BP2 silencing in the transcript half‐life of PIK3CA.

### 2.15 Animal Assays

According to the experimental requirements, KYSE150 cells that had been processed were seeded into T75 culture flasks. Once the cells had conflated, they were digested with trypsin and resuspended in PBS. Healthy male nude mice (*N* = 15, aged 4–6 weeks, Hunan Slyke Jingda Experimental Animal Co. Ltd. Changsha, China) were selected, and the prepared tumor cells were slowly injected subcutaneously into the abdominal region of the mice at a concentration of 5 × 10^6^ cells per suspension. After tumor grafting (8 days postinjection), the health status and tumor growth of the nude mice were observed regularly every 3–5 days. Tumor size was recorded every 5 days, typically by measuring the length and width of the tumor with calipers to calculate the tumor volume. Twenty days after tumor growth, the nude mice were euthanized using xylazine (10 mg/kg, Sigma). Tumor tissue was collected, photographed, and then placed in a fixative solution for subsequent immunohistochemical detection of proliferation markers, as well as tumor weight measurement and western blotting analysis. The study was approved by the Animal Care and Use Committee of Beijing Chao‐Yang Hospital, Capital Medical University.

### 2.16 Immunohistochemistry (IHC) Assay

Tumor tissues were embedded and, then sectioned into slices 4 μm in thickness, which were subsequently dewaxed and rehydrated in xylene and a gradient of ethanol solutions. After washing, the sections were immersed in boiling citrate buffer (pH = 6.2) and heated for 5 min. Following washing, the sections were incubated in 3% H_2_O_2_ in the dark for 10 min. The prepared Ki67 (1:100, Affinity) and PIK3CA antibodies (1:100, Thermo Fisher) were then applied to the sections, which were incubated at 37°C for 1–2 h. After washing, DAB solution was added for staining. The sections were then dehydrated, dried in a gradient of ethanol, and mounted with neutral resin, followed by observation under a microscope.

### 2.17 Statistical Analysis

The statistical data were analyzed using GraphPad 8.0. Quantitative data were expressed as mean ± standard deviation. Comparisons between the two groups were performed using an unpaired *t*‐test. For comparisons among multiple groups, one‐way ANOVA was applied, followed by Tukey's post hoc test. For multiple time‐point data among groups, repeated measures analysis of variance was used. *p* < 0.05 indicated a statistically significant difference.

## 3 Results

### 3.1 METTL3 Expression Was Upregulated in EC Tissues and Cells

**FIGURE 1:**
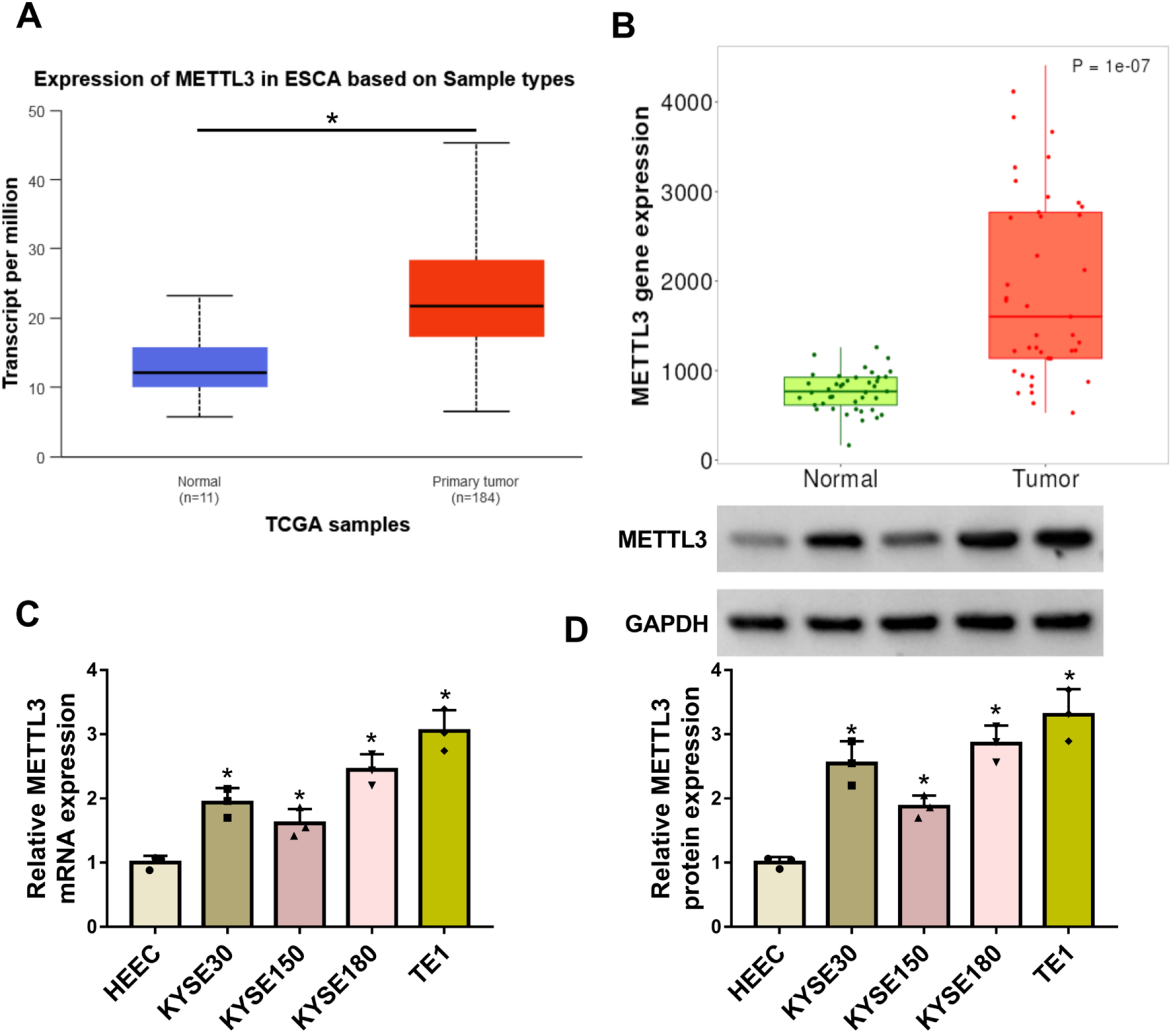
METTL3 expression was upregulated in EC tissues and cells. (A and B) METTL3 expression was analyzed through the TCGA and TNMplot databases in esophageal cancer tissues and normal esophageal tissues. (C and D) METTL3 expression was detected by qRT‐PCR and western blotting assays in HEEC, KYSE30, KYSE150, KYSE180, and TE1 cells. *p < 0.05.

The study analyzed METTL3 expression in EC and normal esophageal tissues through the TCGA and TNMplot databases. As shown in Figure 1A,B, METTL3 expression was significantly increased in tumor tissues when compared with the normal tissues. Moreover, the qRT‐PCR and western blotting assays showed that METTL3 expression was upregulated in EC cell lines, including KYSE30, KYSE150, KYSE180, and TE1 cells, when compared with HEECs, as shown in Figure 1C,D. These data showed a high expression of METTL3 in EC tissues and cells.

### 3.2 METTL3 Knockdown Inhibited the Proliferation, Invasion and Migration of EC Cells and Angiogenesis and Induced Cell Apoptosis

**FIGURE 2:**
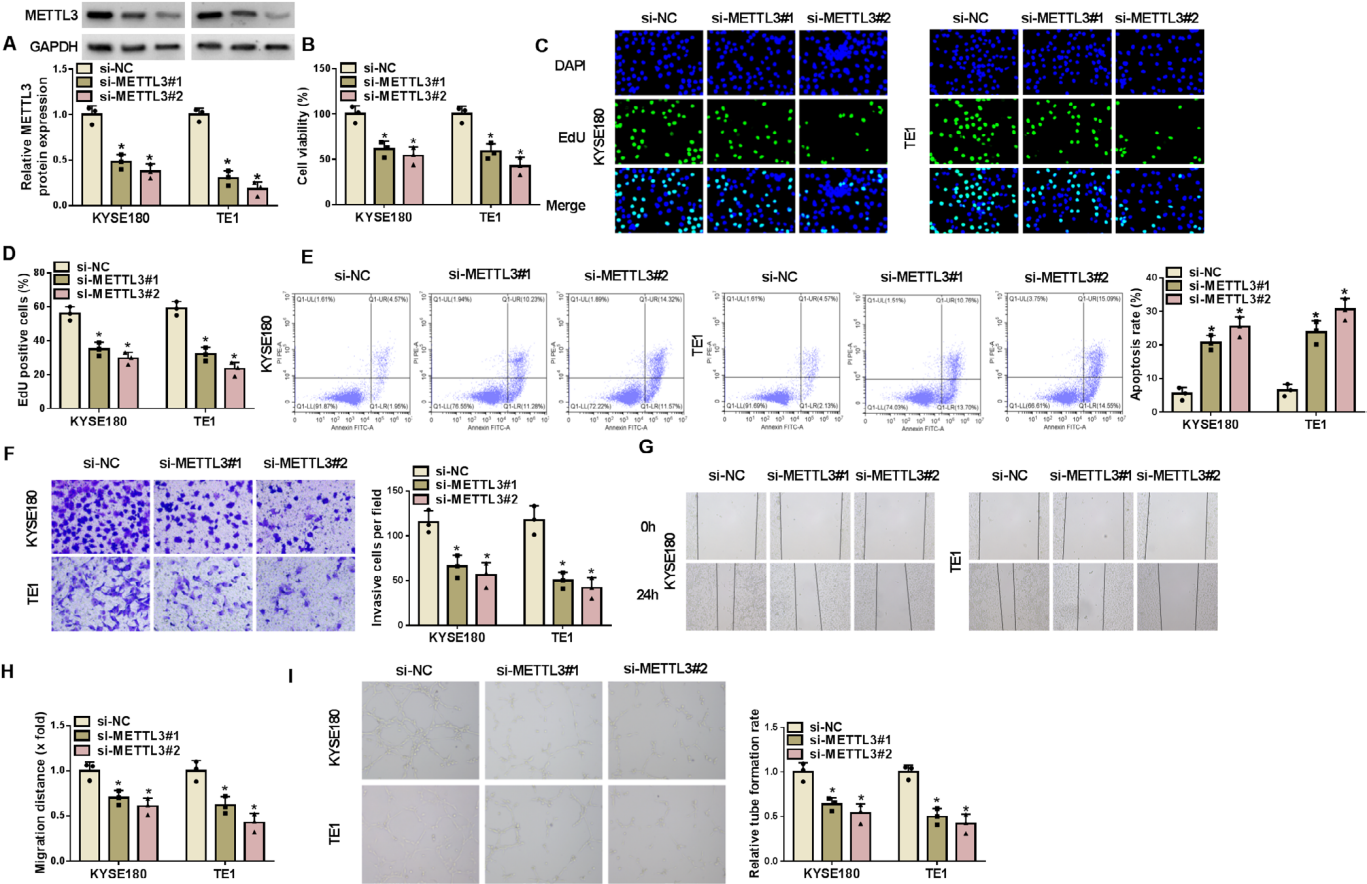
METTL3 knockdown inhibited the proliferation, invasion, and migration of EC cells and angiogenesis and induced cell apoptosis. KYSE180 and TE1 cells were transfected with si‐NC, si‐METTL3#1, and si‐METTL3#2. (A) METTL3 protein expression was detected by western blotting assay. (B) Cell viability was assessed by CCK‐8 assay. (C and D) Cell proliferation was analyzed by EdU assay. (E) Cell apoptosis was analyzed by flow cytometry. (F) Cell invasion was analyzed by transwell invasion assay. (G and H) Wound‐healing assay was performed to analyze cell migration. (I) Angiogenesis was analyzed by tube formation assay. *p < 0.05.

The study then analyzed the effects of METTL3 silencing on the malignant phenotypes of EC cells. To achieve this, the study treated EC cells with METTL3 siRNAs, including si‐METTL3#1 and si‐METTL3#2 and the corresponding control (si‐NC). KYSE180 and TE1 cells were used for the following studies due to higher METTL3 expression in these two cell lines. The data showed that METTL3 expression was significantly downregulated after transfection with these two lines (Figure 2A), indicating the high efficiency of METTL3 knockdown in KYSE180 and TE1 cells. Comparatively, METTL3 silencing inhibited cell viability and proliferation and induced cell apoptosis (Figure 2B–E). Moreover, METTL3 silencing inhibited cell invasion, migration, and angiogenesis (Figure 2F–I). si‐METTL3#2 was used for further analysis based on its more effective effects on the malignant growth of EC cells. Thus, METTL3 depletion inhibited the malignant progression of EC cells.

### 3.3 METTL3 Maintained PIK3CA mRNA Expression in an m6A‐Dependent Manner

**FIGURE 3:**
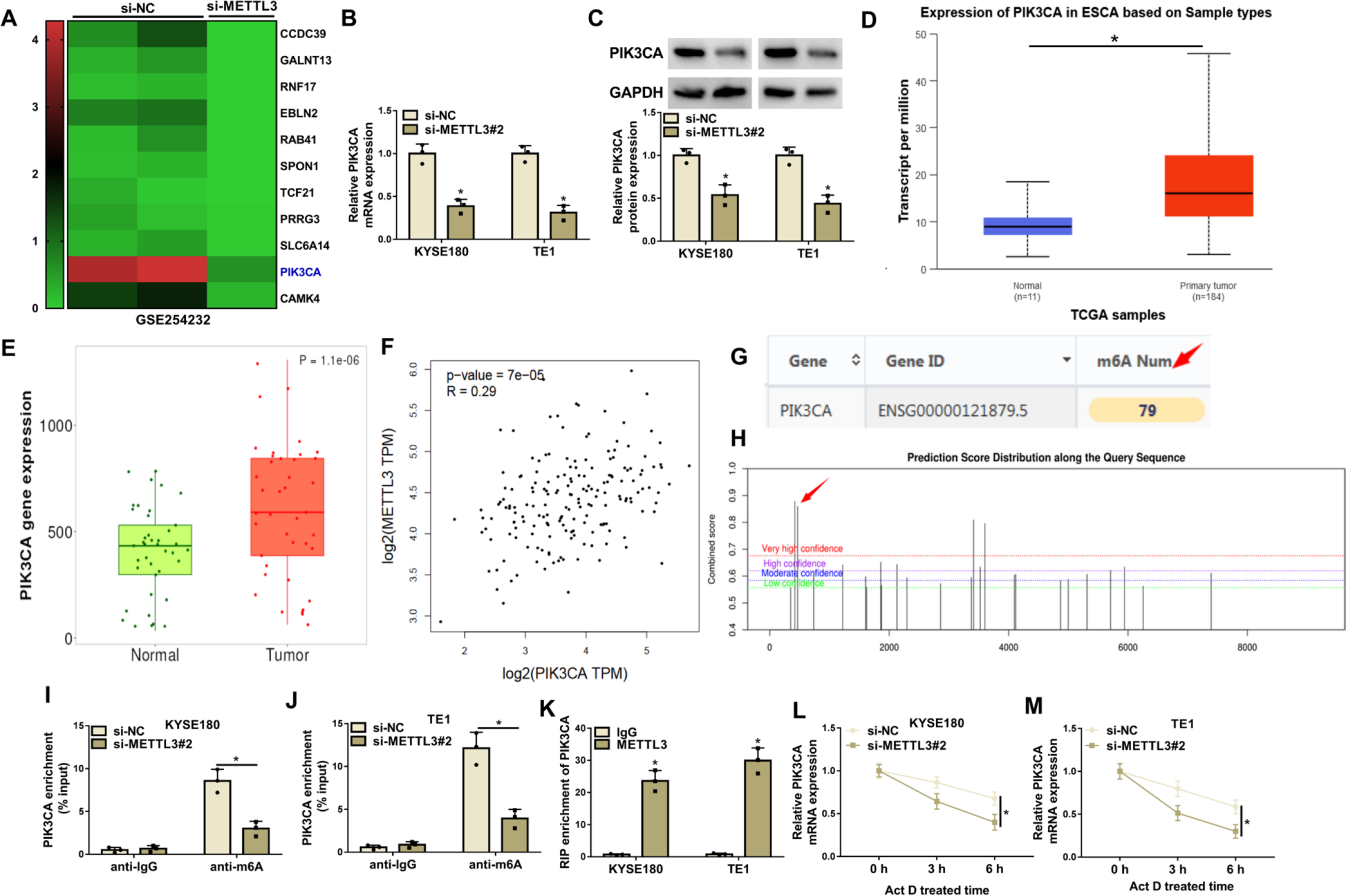
METTL3 maintained PIK3CA mRNA expression in an m6A‐dependent manner. (A) The GEO database (GSE254232) was used to predict the differently expressed genes in EC cells transfected with si‐METTL3 or si‐NC. (B and C) PIK3CA expression was detected by qRT‐PCR and western blotting assays in KYSE180 and TE1 cells transfected with si‐NC or si‐METTL3#2. (D and E) The TCGA and TNMplot databases were used to predict PIK3CA expression in esophageal cancer tissues and normal esophageal tissues. (F) The GEPIA database was used to predict the correlation of METTL3 and PIK3CA expression in esophageal cancer tissues. (G) The RMbase database predicted the presence of m6A methylation modification in PIK3CA. (H) The SRAMP website predicted the existence of methylation modification sites in PIK3CA. (I–M) The MeRIP, RIP, and Actinomycin D assays were used to identify the association of METTL3 and PIK3CA in KYSE180 and TE1 cells. *p < 0.05.

Through the analysis of the GEO database (GSE254232) using EC cells transfected with si‐METTL3 or si‐NC, we discovered that METTL3 depletion inhibited PIK3CA expression (Figure 3A). The result was also confirmed by qRT‐PCR analysis of KYSE180 and TE1 cells transfected with si‐METTL3#2 and si‐NC (Figure 3B). Comparatively, PIK3CA protein expression was downregulated in METTL3‐deficient KYSE180 and TE1 cells (Figure 3C). Through the analysis of the TCGA and TNMplot databases, we discovered a high PIK3CA expression in EC tissues when compared with normal esophageal tissues (Figure 3D,E). In addition, we discovered its positive correlation with METTL3 expression in EC tissues (Figure 3F). Subsequent results showed that the PIK3CA gene might be subjected to m6A methylation modification. For example, there were m6A methylation modification and methylation modification sites in PIK3CA (Figure 3G,H). Moreover, the binding affinity of the m6A antibody to PIK3CA was weakened after METTL3 silencing (Figure 3I,J). As presented in Figure 3K, the METTL3 exhibited a heightened capacity for binding to PIK3CA in KYSE180 and TE1 cells. Further, the transcript half‐life of PIK3CA was shortened after METTL3 silencing in KYSE180 and TE1 cells (Figure 3L,M). Thus, METTL3 stabilized PIK3CA through the m6A methylation modification.

### 3.4 METTL3 Maintained PIK3CA mRNA Stability in an IGF2BP2‐Dependent Manner

**FIGURE 4:**
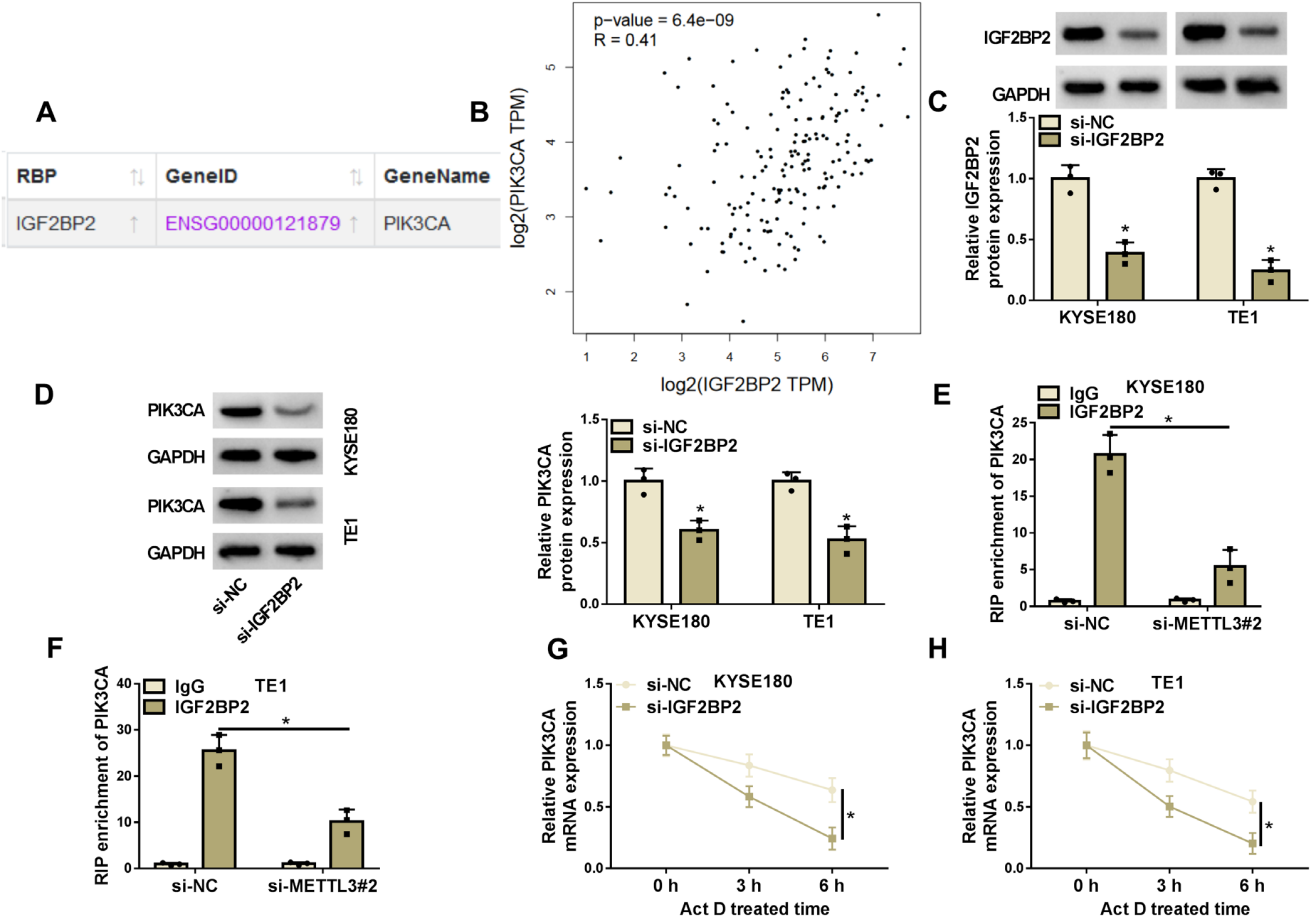
METTL3 maintained PIK3CA mRNA stability in an IGF2BP2‐dependent manner. (A) The ENCORI database was used to predict the association of IGF2BP2 and PIK3CA. (B) The GEPIA database was used to analyze the correlation of IGF2BP2 and PIK3CA in esophageal cancer tissues. (C and D) The protein expression of IGF2BP2 and PIK3CA was analyzed by western blotting assays in KYSE180 and TE1 cells transfected with si‐NC or si‐IGF2BP2. (E and F) The RIP assay was performed to analyze the association of IGF2BP2 with PIK3CA in KYSE180 and TE1 cells transfected with si‐NC or si‐METTL3#2. (G and H) Actinomycin D assay was used to detect the transcript half‐life of PIK3CA in KYSE180 and TE1 cells transfected with si‐NC or si‐IGF2BP2. *p < 0.05.

Through the prediction of the ENCORI database, we discovered that IGF2BP2 was an RNA‐binding protein of PIK3CA (Figure 4A). Moreover, IGF2BP2 showed a positive correlation with PIK3CA expression in EC tissues (Figure 4B). The study then transfected IGF2BP2 siRNA and its control si‐NC into KYSE180 and TE1 cells. As shown in Figure 4C, IGF2BP2 protein expression was significantly downregulated after transfection with si‐IGF2BP2, indicating the high efficiency of IGF2BP2 knockdown. Subsequently, we discovered that IGF2BP2 depletion significantly inhibited PIK3CA protein expression in KYSE180 and TE1 cells (Figure 4D). Comparatively, the binding affinity of IGF2BP2 to PIK3CA was weakened after METTL3 silencing (Figure 4E,F). The transcript half‐life of PIK3CA was shortened in KYSE180 and TE1 cells after transfection with si‐NC or si‐IGF2BP2 (Figure 4G,H). Moreover, the simultaneous knockdown of METTL3 and IGF2BP2 yielded the most significant effect (Figure S1). Thus, METTL3 might induce the m6A methylation modification of PIK3CA by interacting with IGF2BP2.

### 3.5 PIK3CA Overexpression Attenuated METTL3 Silencing‐Induced Effects on the Malignant Growth of KYSE180 and TE1 Cells

**FIGURE 5:**
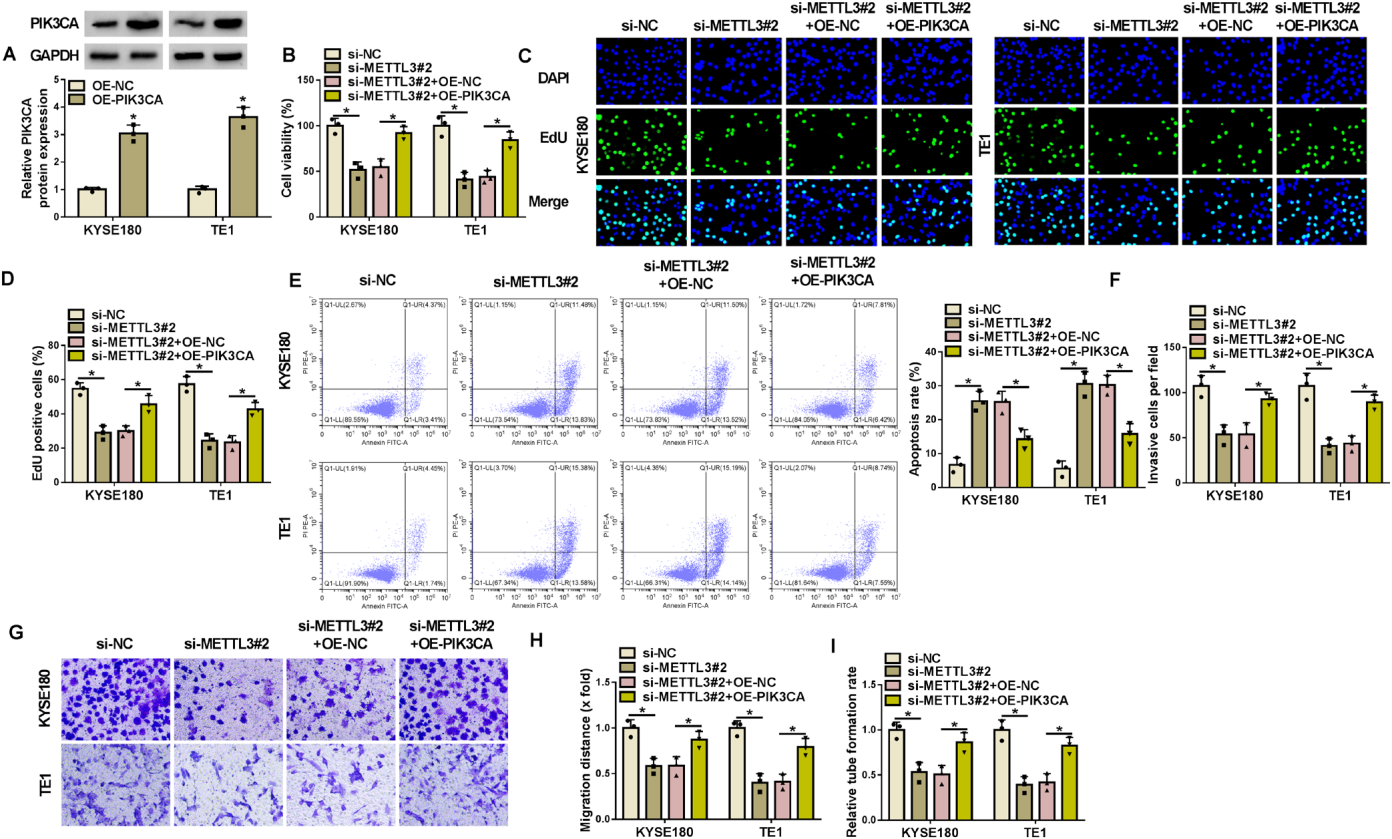
PIK3CA overexpression attenuated METTL3 silencing‐induced effects on the malignant growth of KYSE180 and TE1 cells. (A) The efficiency of PIK3CA overexpression was analyzed by western blotting assay. KYSE180 and TE1 cells were transfected with si‐NC, si‐METTL3#2, si‐METTL3#2 + OE‐NC, or si‐METTL3#2 + OE‐PIK3CA. (B) Cell viability was assessed by CCK‐8 assay. (C and D) Cell proliferation was analyzed by EdU assay. (E) Cell apoptosis was analyzed by flow cytometry. (F and G) Cell invasion was analyzed by transwell invasion assay. (H) Wound‐healing assay was performed to analyze cell migration. (I) Angiogenesis was analyzed by tube formation assay. *p < 0.05.

The study subsequently validated whether the effects of METTL3 on the malignant phenotypes of KYSE180 and TE1 cells involved the regulation of PIK3CA. To achieve this, the study transfected METTL3 siRNA, PIK3CA overexpression plasmid, and the respective controls into KYSE180 and TE1 cells. The efficiency of PIK3CA overexpression was high, and the results were confirmed by western blotting assay (Figure 5A). Then, METTL3 silencing inhibited cell viability and proliferation and induced cell apoptosis, whereas these effects were relieved after PIK3CA overexpression (Figure 5B–E). METTL3 silencing inhibited cell invasion, migration, and angiogenesis; however, these effects were attenuated after transfection with PIK3CA overexpression plasmid (Figures 5F–I and S2A,B). Thus, METTL3 silencing inhibited the malignant growth of EC cells by regulating the PIK3CA pathway.

### 3.6 METTL3 Silencing Inactivated the AKT Pathway by Regulating PIK3CA Expression

**FIGURE 6:**
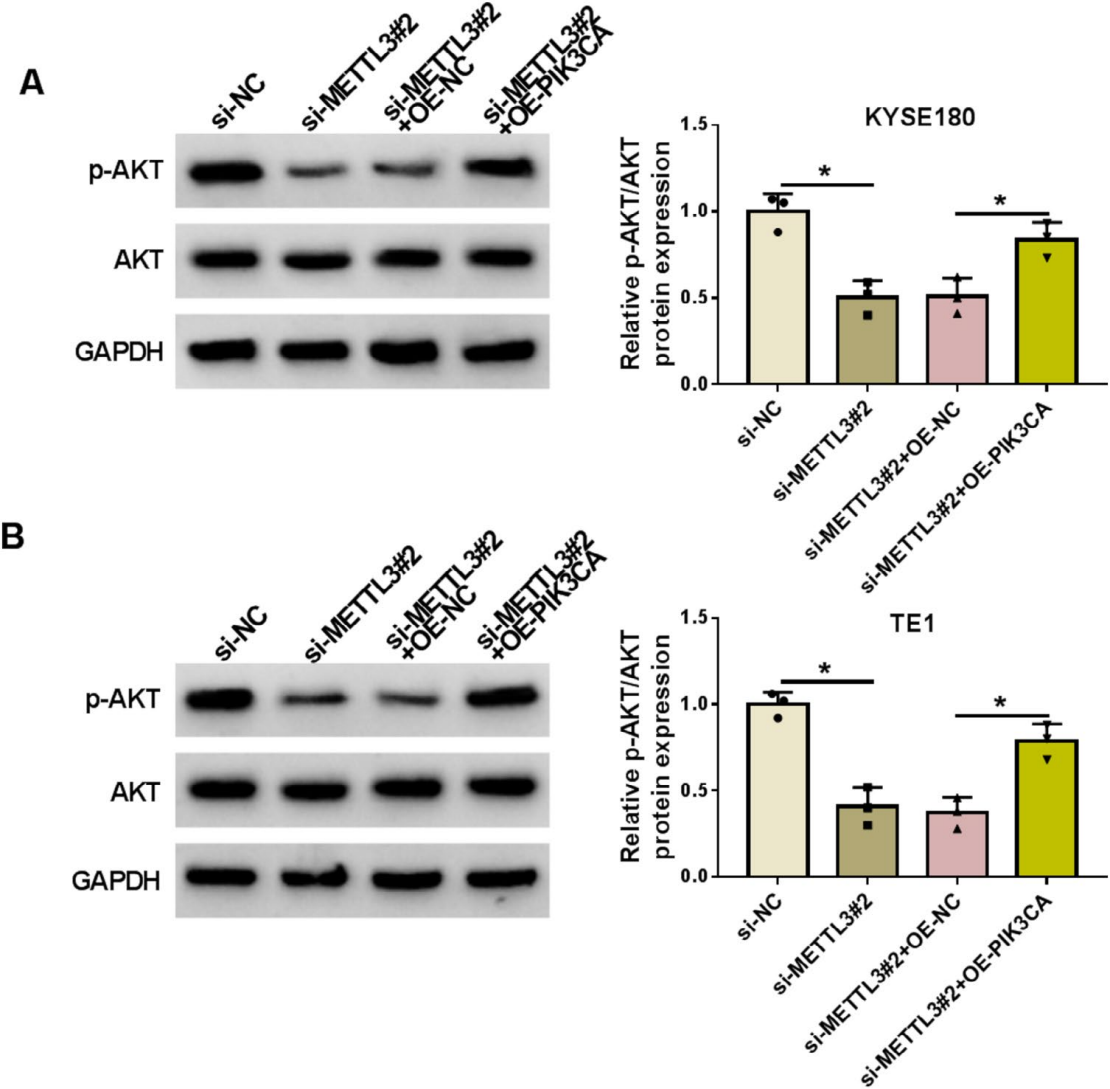
METTL3 silencing inactivated the AKT pathway by regulating PIK3CA expression. (A and B) KYSE180 and TE1 cells were transfected with si‐NC, si‐METTL3#2, si‐METTL3#2 + OE‐NC, or si‐METTL3#2 + OE‐PIK3CA. The protein expression of p‐AKT and AKT was analyzed by western blotting assay. *p < 0.05.

The study subsequently analyzed the effects of METTL3 and PIK3CA on the AKT pathway. As shown in Figure 6A,B, the value of p‐AKT/AKT was inhibited after METTL3 silencing, whereas the effect was attenuated after PIK3CA overexpression.

### 3.7 METTL3 Silencing Inhibited Tumor Formation by Regulating PIK3CA Expression

**FIGURE 7:**
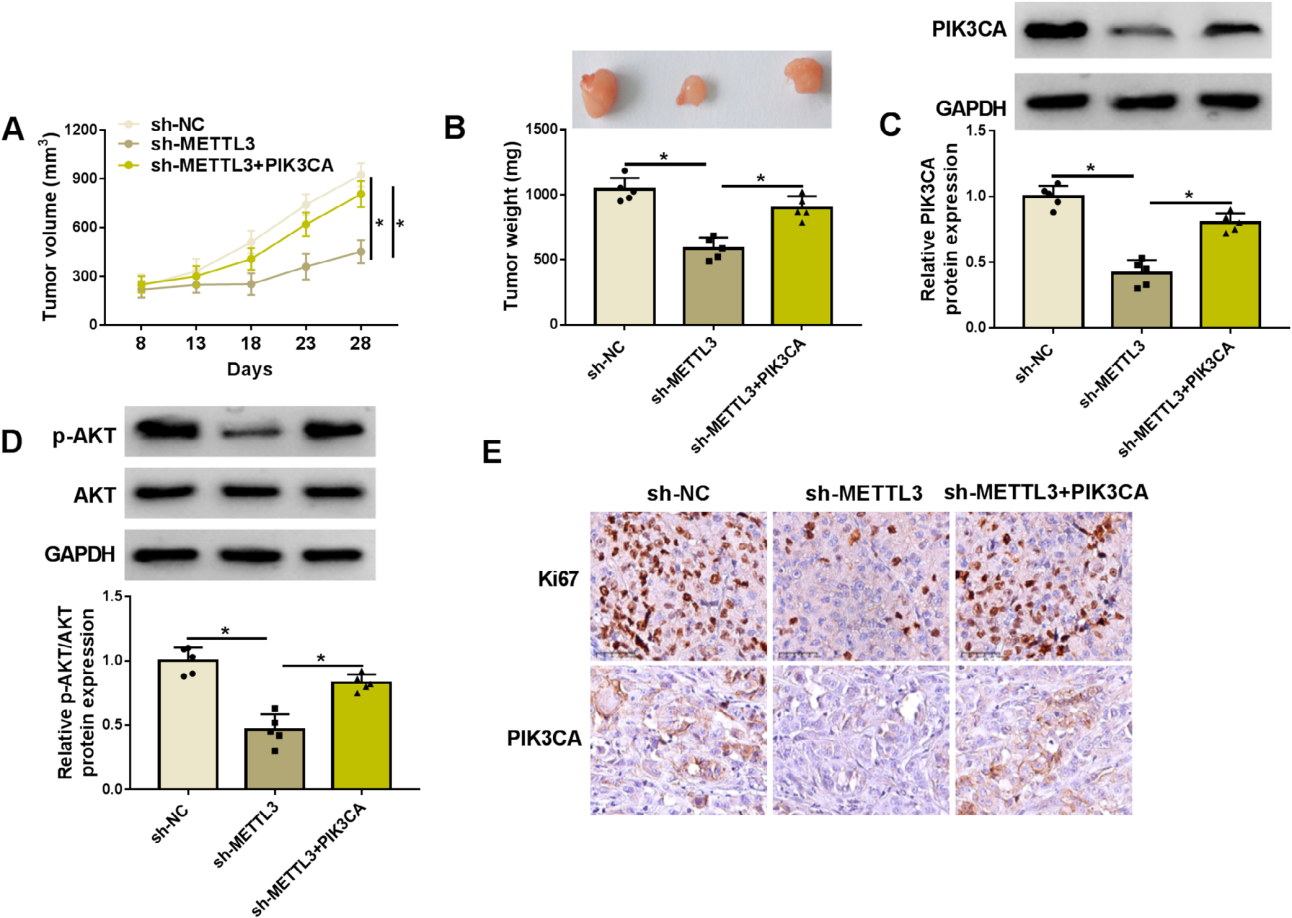
METTL3 knockdown inhibited the malignant growth of EC cells by regulating PIK3CA expression. The study injected nude mice with KYSE150 cells expressing sh‐NC, sh‐METTL3, or sh‐METTL3 in combination with a PIK3CA overexpression plasmid. After 8 days, tumor volume was analyzed every 5 days for four cycles (A). After 28 days, the forming tumors were harvested for the analysis in tumor weight (B) and the protein expression of PIK3CA, p‐AKT, AKT, and Ki67 (C–E). *p < 0.05.

The study further performed the xenograft mouse model assay to validate the in vitro data regarding the effects of METTL3 and PIK3CA on the malignant growth of EC cells. To end this, the study injected nude mice with KYSE150 cells expressing sh‐NC, sh‐METTL3, or sh‐METTL3 in combination with a PIK3CA overexpression plasmid. After detecting the volume and weight of the resulting tumors, we found that the silencing of METTL3 led to a significant delay in tumor growth, with the effect becoming evident from Day 8 and persisting through Day 28, whereas the treatment in combination with PIK3CA overexpression relieved the effects (Figures 7A,B and S3). Moreover, we discovered that the expression of PIK3CA and Ki67 as well as the value of p‐AKT/AKT were reduced in the resulting tumors from the METTL3 silencing group; however, these effects were counteracted in these tumors resulting from the combination treatment group (Figure 7C–E). Thus, METTL3 knockdown inhibited the malignant growth of EC cells by regulating PIK3CA expression.

## 4 Discussion

EC is one of the world's deadliest and fastest‐growing cancers, typically characterized by symptoms such as difficulty in swallowing, unintentional weight loss, and others [19]. Despite significant advancements in the treatment of EC, the prognosis remains poor [20]. This necessitates the development of new therapeutic approaches. METTL3, as a methyltransferase, exhibits oncogenic properties by depositing m6A modifications on critical transcripts, promoting the development and progression of various cancers, including hematologic malignancies and solid tumors, and holds potential for cancer therapy [10]. Therefore, investigating METTL3's role in EC progression could provide a theoretical basis for the treatment of EC. The present work revealed that METTL3/IGF2BP2 maintained PIK3CA expression to activate the AKT pathway, thus promoting EC progression.

METTL3 has been reported to be upregulated in EC tissues and cells [21] and can promote the growth and motility of EC cells by inducing the expression of NOTCH1 and subsequent activation of the NOTCH1 pathway [11]. Moreover, it was reported that METTL3 promoted EC cell migration and invasion by inducing GLS2 expression [22]. We also identified the high expression of METTL3 in EC tissues and cells and its promoting effects on EC cell proliferation, migration and invasion as well as tumor formation. In addition, we discovered that METTL3 inhibited EC cell apoptosis and promoted tube formation. Importantly, our data demonstrated that METTL3 induced PIK3CA expression in an m6A‐dependent manner. IGF2BP2 is an m6A‐binding protein that has been reported in EC progression. For example, IGF2BP2 upregulated circRUNX1 to promote EC cell proliferation and metastasis by regulating miR‐449b‐5p/FOXP3 pathway [23]. IGF2BP2 increased OCT4 mRNA expression to promote the stem cell‐like property of EC cells [24]. We presented compelling evidence that METTL3's influence on PIK3CA mRNA stability was mediated through the RNA‐binding protein IGF2BP2. While our data clearly showed that IGF2BP2's binding to PIK3CA mRNA was influenced by METTL3, it was essential to address whether this interaction was unique to PIK3CA or if it represented a broader pattern of regulation. IGF2BP2 is known to bind a variety of mRNAs and is involved in the regulation of mRNA stability and translation for multiple target genes. Therefore, it is plausible that METTL3‐induced m6A modifications may affect the binding of IGF2BP2 to other mRNAs as well. To determine the specificity of IGF2BP2's action, future studies could investigate the binding patterns of IGF2BP2 to a panel of mRNAs in the presence and absence of METTL3. Additionally, it would be informative to assess whether other m6A‐binding proteins are similarly affected by METTL3 knockdown, which would provide a more comprehensive understanding of the regulatory network involving m6A methylation and RNA‐binding proteins.

Our data showed a high PIK3CA expression in EC tissues, and it is promoting effects on EC cell proliferation, migration, invasion, and angiogenesis. Moreover, we discovered its inhibiting effect on EC cell apoptosis. The METTL3 silencing‐induced inhibitory effects on the malignant growth of EC cells were also counteracted after PIK3CA overexpression in vitro and in vivo, indicating the involvement of the METTL3/PIK3CA axis in EC progression. In various malignant tumors, the PI3K/AKT signaling pathway is activated, which plays a crucial role in regulating malignant tumor cells [25, 26, 27]. PIK3CA, which encodes the catalytic subunit of PI3K, can trigger the activation of PI3K. Once PI3K is activated, it can further activate downstream effector AKT through a series of enzymatic reactions. AKT can phosphorylate a variety of transcription factors, upregulating anti‐apoptotic and apoptosis‐inhibitory genes, thus promoting cell survival and proliferation, ultimately leading to the development and progression of tumors [28]. In the development of EC, it has been reported the promoting effect of the AKT pathway in tumor progression [29, 30, 31]. Our data showed that PIK3CA activated the AKT pathway in EC cells, and METTL3 silencing inactivated the AKT pathway by regulating PIK3CA. Thus, the METTL3/PIK3CA/AKT pathway was responsible for EC progression.

However, the study is based on in vitro and in vivo experiments, which may not fully reflect the complex physiological and pathophysiological conditions within the human body. Therefore, the findings may require further validation in clinical settings. In addition, the study identifies a molecular mechanism involving m6A modification and IGF2BP2, but the precise clinical implications and the potential therapeutic window for targeting these molecules remain to be determined.

Taken together, METTL3/IGF2BP2 promoted the malignant progression of EC by inactivating the PIK3CA/AKT pathway. The finding provides a novel insight into EC development. This insight could lead to the identification of new therapeutic targets for the disease.

## Author Contributions

Xinmeng Guo conducted the experiments and drafted the manuscript. Meng Yang and Anqi Huang contributed to the collection of documents. Ya'nan Qi revised the editable manuscript. Jiaqi Chen contributed to the methodology and plotted the figures. Mulan Jin contributed to the study concept and design. All authors read and approved the final manuscript. Mulan Jin was the corresponding author.

## Conflicts of Interest

The authors declare no conflicts of interest.

## Supporting information


**Figure S1.** The effect of simultaneous knockdown of METTL3 and IGF2BP2 on PIK3CA mRNA stability. *p < 0.05.

**Figure S2.** The representative images of Figure 5H,I.

**Figure S3.** The representative images of mice in sh‐NC group, sh‐METTL3 group, and sh‐METTL3 + PIK3CA group.

## Data Availability

Data are available from the corresponding author upon reasonable request.
