# Supplementary figures and images for: METTL3/IGF2BP2 Promotes the Malignant Progression of Esophageal Cancer by Activating the PIK3CA/AKT Pathway
Source: Thorac Cancer. 2025 Feb 20;16(4):e70022. doi: 10.1111/1759-7714.70022 (PMC11842509; doi:10.1111/1759-7714.70022)

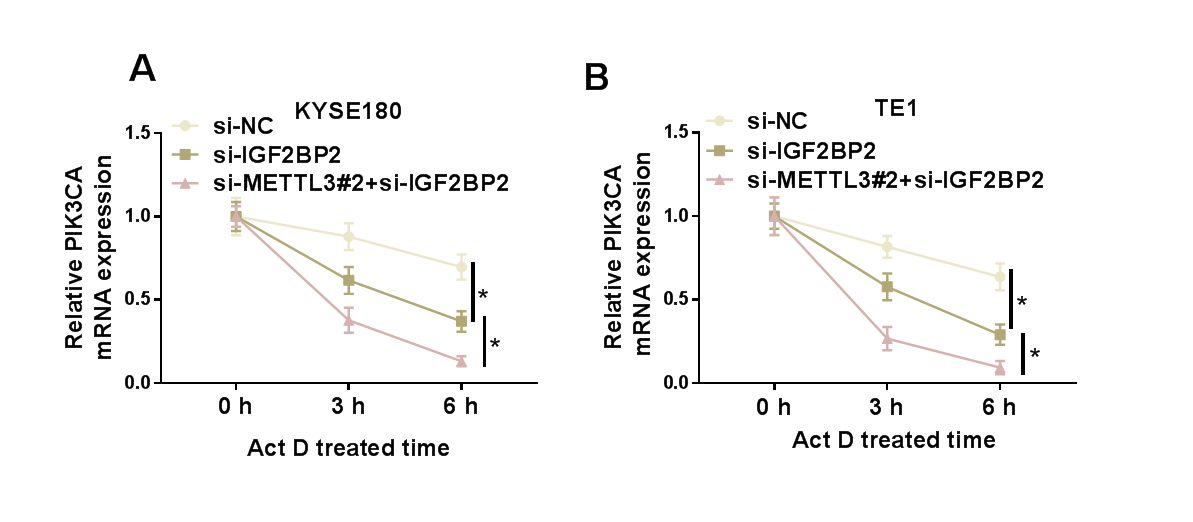

Supplement: Supplementary file 1 — Figure S1. The effect of simultaneous knockdown of METTL3 and IGF2BP2 on PIK3CA mRNA stability. *p < 0.05. [file TCA-16-e70022-s001.tif]

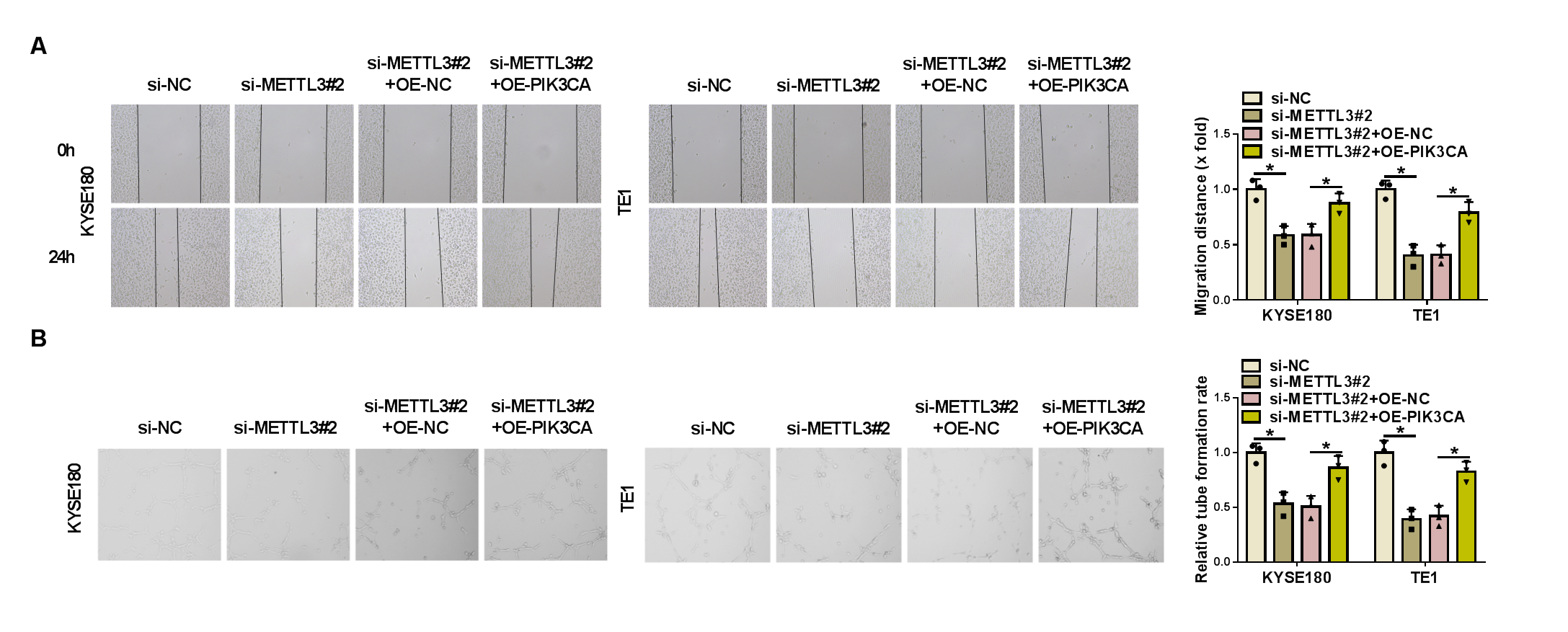

Supplement: Supplementary file 2 — Figure S2. The representative images of Figure 5H,I. [file TCA-16-e70022-s002.tif]

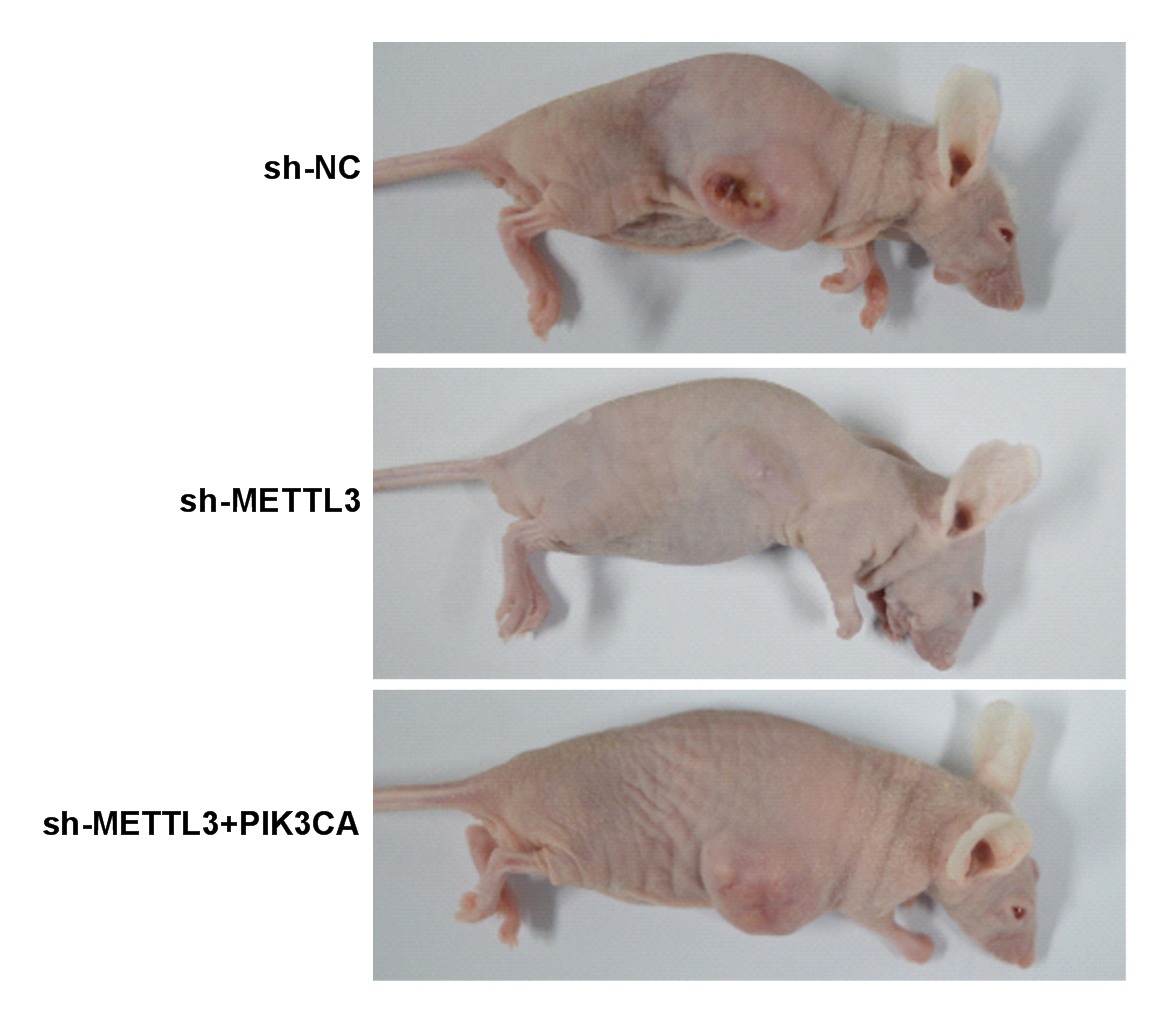

Supplement: Supplementary file 3 — Figure S3. The representative images of mice in sh‐NC group, sh‐METTL3 group, and sh‐METTL3 + PIK3CA group. [file TCA-16-e70022-s003.tif]
